# Treatment of Anterior Sternoclavicular Joint Dislocation with Acromioclavicular Joint Hook Plate

**DOI:** 10.1111/os.12422

**Published:** 2019-02-06

**Authors:** Yan‐zhen Qu, Tian Xia, Guo‐hui Liu, Wu Zhou, Bo‐bin Mi, Jing Liu, Xiao‐dong Guo

**Affiliations:** ^1^ Department of Orthopaedics, Union Hospital, Tongji Medical College Huazhong University of Science and Technology Wuhan China

**Keywords:** Acromioclavicular joint, Hook plate, Physical function, Sternoclavicular joint dislocation

## Abstract

**Objective:**

To evaluate the safety and efficacy of using acromioclavicular joint hook plates for the treatment of anterior sternoclavicular joint dislocation.

**Methods:**

Ten patients who suffered anterior sternoclavicular joint dislocation were retrospectively analyzed, and underwent acromioclavicular joint hook plate surgeries from January 2015 to May 2017. There were 7 male and 3 female patients, with a mean age of 43.6 years. According to the American Shoulder and Elbow Society (ASES) scoring system, the preoperative physical function had a mean of 83.5.

**Results:**

Reduction and fixation were performed with hook plates in all 10 patients. All patients were followed up, with a mean duration of 16.9 months. There were no complications, no wound infections, and no plate or screw breakages. Movement of the shoulder girdle was improved in all patients. According to the ASES scoring system, the postoperative physical function had a mean of 94.8.

**Conclusion:**

The acromioclavicular joint hook plate demonstrates safety and efficacy for the treatment of anterior sternoclavicular joint dislocation. However, there are still some deficiencies that need to be improved.

## Introduction

The sternoclavicular joint is a diarthrodial saddle type synovial joint. Between the upper extremity and the axial skeleton, the sternoclavicular joint is the only bony articulation^1^, ^2^, ^3^. The sternoclavicular joint is inherently unstable because the medial clavicular surface articulates with its corresponding articular surface on the manubrium sterni less than 50%^3^, ^4^. Any movement of the shoulder girdle could cause some motion of the sternoclavicular joint. The clavicle elevates approximately 4° for every 10° of arm forward flexion^5^. When there are some combined movements, the clavicle can rotate up to 40° along its longitudinal axis. Due to the force at the shoulder girdle, the huge movement of the shoulder girdle can result in the dislocation of the sternoclavicular joint anteriorly or posteriorly. Dislocation occurs easily in patients with a short clavicle, which results in more torque.

The stability of the sternoclavicular joint depends mainly on the intrinsic and extrinsic ligament structures surrounding the joint, and this makes the sternoclavicular joint the constricted joint^3^, ^4^. The costoclavicular ligament is divided into anterior fasciculus, which resists superior rotation and lateral displacement, and posterior fasciculus, which resists inferior rotation and medial displacement^1^. Besides the costoclavicular ligament, the other ligaments surrounding the sternoclavicular joint could also aid stability of the joint.

Due to the stable structures surrounding the sternoclavicular joint, sternoclavicular joint dislocations are infrequent, and represent only 3% of all dislocations in the shoulder girdle in the clinic^6^, ^7^. Because of the different injury mechanisms, dislocation directions, and clinical manifestations, sternoclavicular joint dislocation can be divided into anterior dislocation and posterior dislocations. The lateral compressive force which effects the shoulder girdle could cause rupture of the anterior capsule and the costoclavicular ligament, and result in anterior dislocation of the sternoclavicular joint^4^.

The incidence of anterior dislocation is almost 90% in all sternoclavicular joint dislocations^8^. Because the stable structures around the dislocated sternoclavicular joint are broken, manual reduction of the anterior sternoclavicular joint dislocation is difficult and redislocation occurs frequently after reduction.

Because some important tissues, such as the pleura, lung, mediastinum and trachea, are just behind the sternoclavicular joint, high risks exist in the surgical treatment of sternoclavicular dislocation. Surgical methods such as Kirschner wires, FiberWire, two screws and a strong suture, T‐plates, and locking compression plates have been used in the treatment of sternoclavicular dislocation, but there are still complications, including limited movement of the shoulder girdle, redislocation, vascular and nerve rupture, pleura rupture, vital organ injuries, and displacement and breakage of plates and screws^9^, ^10^, ^11^, ^12^, ^13^. These serious complications require that the internal fixation has stability, micromotion, and fewer screws, which could avoid the severe consequences of these complications. However, there is still no ideal surgical method.

To provide micromotion and stability, and to avoid the injury of important organs by screws, we used an acromioclavicular joint hook plate for the treatment of anterior sternoclavicular joint dislocation. The purpose of the present study is to evaluate the safety and efficacy of using an acromioclavicular joint hook plate for the treatment of sternoclavicular joint dislocation, and to present the functional outcomes, in a series of 10 patients, at a minimum of 10 months of follow‐up.

## Methods

### *General Data*

From January 2015 to May 2017, 10 patients with anterior sternoclavicular joint dislocation were admitted and surgically treated with acromioclavicular joint hook plates at our department. The Committee on Research Ethics of the Union Hospital approved this study. Exclusion criteria included that: (i) the patient had a brain injury or serious underlying chronic illness and, therefore, could not suffer the risk of surgery and anesthesia; and (ii) the patient demanded the conservative treatment even if closed reduction was pointless. There were 7 male and 3 female patients, with a mean age of 43.6 years. Two patients suffered from bilateral dislocations of the sternoclavicular joints, 8 patients suffered from unilateral dislocation, and 1 of the 10 patients had an old dislocation (more than 3 weeks). Three patients had rib fractures; 1 patient had a tibia fracture; and 1 patient had a clavicle fracture on the other side. Injury mechanism: 6 patients had car accidents, 3 patients fell from a high place, and 1 patient was injured by a crashing object.

All patients underwent the standard preoperative assessment, including preoperative history, physical examination, chest X‐ray, and computed tomography (CT) scan. Closed reduction was attempted for all the patients but failed, so surgery was undertaken. The interval between injury and surgery was from 3 to 7 days for the 9 acute patients.

### *Surgical Technique*

**Figure 1:**
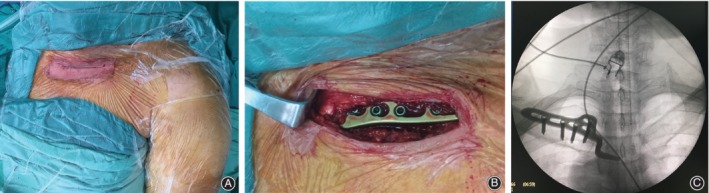
Hook plate fixation in a patient with left anterior sternoclavicular joint dislocation. (A) An anterosuperior straight incision was extended from the medial clavicle to the midsuperior aspect of the sternal manubrium. (B) The lever effect was taken to press the medial end of the clavicle down for reduction by the hook plate. (C) The intraoperative fluoroscopy showed that the dislocation was reduced successfully by the hook plate.

All patients were positioned supine on the operating table, and underwent general anesthesia. An anterosuperior straight incision was extended from the medial clavicle to the midsuperior aspect of the sternal manubrium, and the sternoclavicular joint, the sternal manubrium, and the medial clavicle were exposed. The incarcerated soft tissue of the sternoclavicular joint was cleaned, and the broken sternoclavicular joint cartilage plate was replaced or cleaned. The pointed end of the acromioclavicular joint hook plate was inserted into the dorsal osteal face of the sternal manubrium, and the lever effect was taken to press the medial end of clavicle down for reduction (Fig. 1). When the dislocation was successfully replaced by the hook plate, three or four screws were fixed in the clavicle through the plate. The broken anterior sternoclavicular ligament and the costoclavicular ligament were repaired with absorbable sutures.

### *Postoperative Management*

In the first 4 weeks, the shoulder was immobilized in a sling, and easy exercises such as pendulum exercises in the glenohumeral joint were allowed, but not over 90° of abduction. After 4 weeks, the range of motion could be increased according to the clinical course. Sporting activities were to be avoided in the first 12 weeks. The hook plate could be removed 12 months postoperatively according to the clinical course.

### *Follow up*

All the patients were followed up with a mean duration of 16.9 months (range, 10–24 months). Chest X‐rays and motion range measurement of the glenohumeral joint were taken on the first postoperative day, after 4, 8, and 12 weeks, and then taken once half yearly. The American Shoulder and Elbow Society (ASES) scoring system was used to assess the preoperative and postoperative physical function and ability of patients.

## Results

### *General Data*

In this study, 8 patients underwent unilateral operations and 2 patients underwent bilateral operations. The mean operative blood loss was 45 mL (range, 30–90 mL), and the mean operative time was 0.8 h (range, 0.4–1.5 h). There were no respiratory or circulatory issues in the operation. There was no postoperative wound infection, and there were no complications such as joint redislocation, vascular rupture, pleura rupture, or vital organ injury. The associated injuries were treated effectively. There was no plate breakage or screw breakage at final follow‐up.

### *X‐rays and Motion Range Measurement*

The postoperative X‐rays showed that all the dislocated joints were reduced successfully, the location and angle of the plates were suitable, and no redislocation appeared in the follow‐up period. There was no osteolysis observed in the sternum for all the patients.

### *Physical Function*

**Figure 2:**
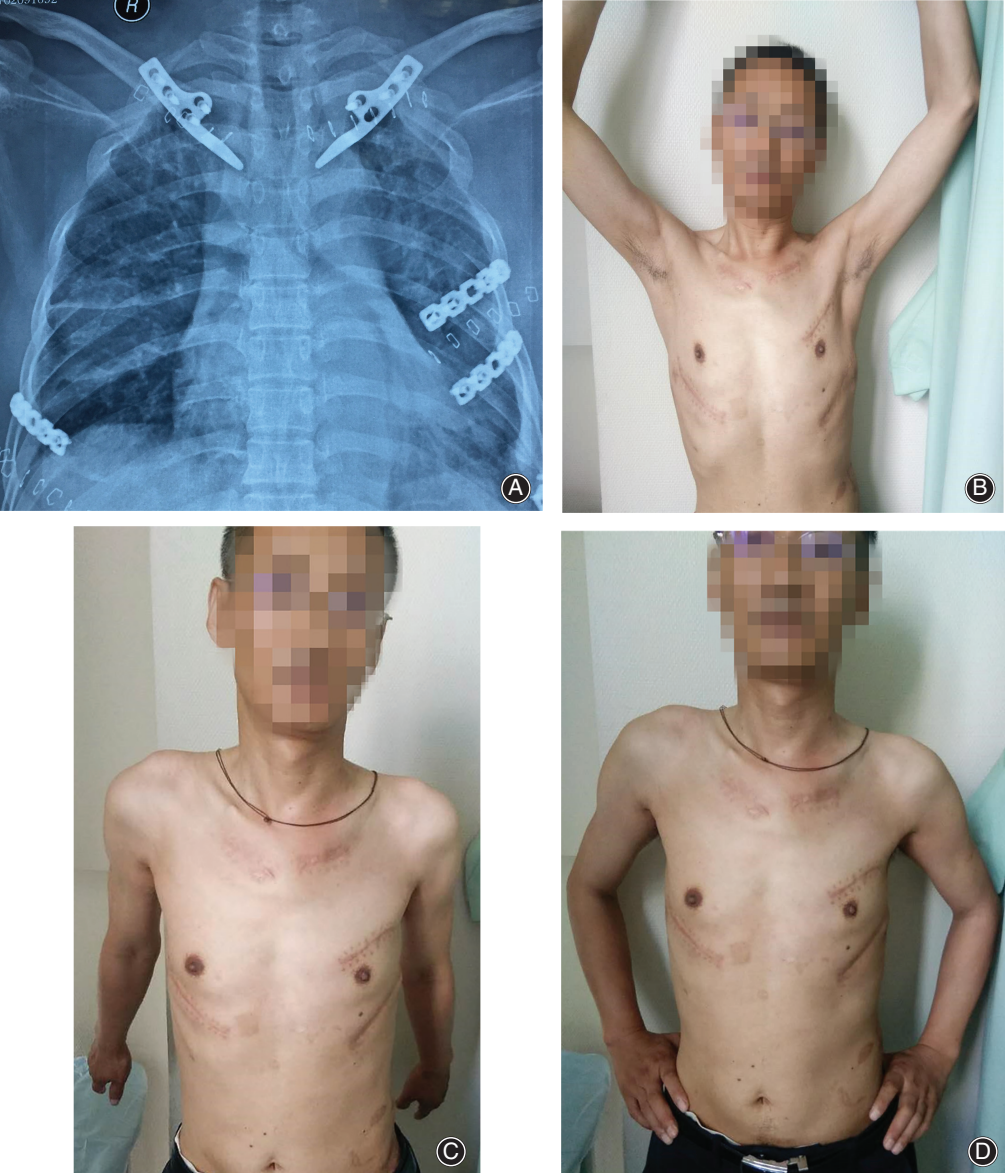
A patient with bilateral dislocation of the sternoclavicular joints and rib fratures. (A) The postoperative X‐ray image showed the well reduction by hook plates. (B–D) Functional outcome 6 months after surgery.

**Figure 3:**
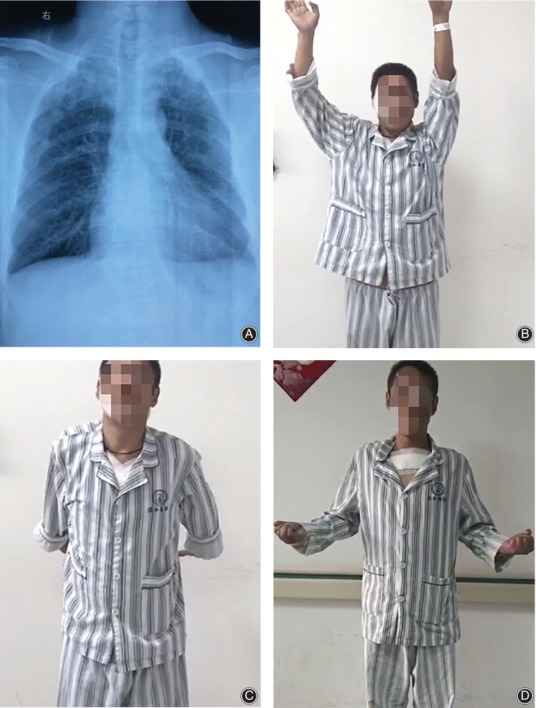
After 17 months, the internal fixations of the patient in Fig. 2 were all removed. (A) The postoperative X‐ray image showed that the sternoclavicular joints were still stable after the removal of internal fixation. (B–D) Functional outcome 17 months after the first surgery.

The postoperative abduction angle of the glenohumeral joint had a mean of 164.3° (range, 153°–172°) and the angles of two glenohumeral joints were less than 160°. The posterior extension angle of the glenohumeral joint had a mean of 39.9° (range, 30°–44°). The forward elevation had a mean of 147° (range, 135°–165°). The horizontal extension had a mean of 24.5° (range, 21°–29°) (Figs 2 and 3).

According to the ASES scoring system, the postoperative physical function had a mean of 94.8, which was better than the preoperative function, which had a mean of 83.5 (Table 1).

**Table 1:** General data, American Shoulder and Elbow Society scoring system before and after operation, and the postoperative movements of glenohumeral joints of all 10 patients

No.	Sex	Age (years)	Pre ASES	Post ASES	Follow‐up (months)	Post abduction angle (°)	Post extension angle (°)	Post forward elevation (°)	Post horizontal extension (°)
1	M	49	69	89	18	160(R)/153(L)	44(R)/38(L)	155(R)/140(L)	26(R)/22(L)
2	M	28	75	92	22	172(R)/168(L)	40(R)/43(L)	165(R)/148(L)	26(R)/22(L)
3	M	64	81	95	10	170	41	158	25
4	M	30	86	98	19	163	40	142	25
5	M	53	83	96	24	169	44	149	29
6	M	40	86	96	14	164	36	141	21
7	M	37	88	98	11	168	43	144	26
8	F	38	82	96	15	160	41	143	27
9	F	42	86	96	17	168	39	144	23
10	F	55	79	92	19	157	30	135	22

## Discussion

As a diarthrodial saddle type synovial joint, the sternoclavicular joint is inherently unstable, and it is also the only bony articulation between the axial skeleton and the upper extremity^1^, ^2^, ^3^. The ligaments surrounding the sternoclavicular joint guarantee the stability of the joint^2^. When there is a lateral compressive force effect on the shoulder girdle, it can cause rupture of the anterior capsule and part of the costoclavicular ligament, which results in the anterior dislocation of the sternoclavicular joint^8^. Due to the broken ligaments with a high energy injury, redislocation happens frequently after manual reduction, and the conservative treatment shows poor efficacy in sternoclavicular joint dislocation patients, for instance with progressive pain limiting the movement of the shoulder girdle and decreasing quality of life^14^.

There are many important thoracic structures, such as the trachea, the esophagus, brachiocephalic veins, the brachiocephalic artery, and the brachial plexus, located posteriorly to the sternoclavicular joint^15^, ^16^. The complex structures surrounding the sternoclavicular joint mean that not only can the sternoclavicular joint dislocation cause trauma (e.g. rupture of blood vessel, nerve injury, mediastinum organs injury, pleura rupture, and lung rupture), but also fewer screws should be used, especially on the sternal manubrium, in the reduction of dislocation to prevent accidental injury by internal fixation^3^.

Surgical technologies including Kirschner wires, FiberWire, two screws and a strong suture, T‐plate, and locking compression plate, have been reported on for the treatment of steroclavicular joint dislocation^5^, ^6^, ^7^, ^8^, ^9^. For the methods using wires, a significant risk to mediastinal structures with wire migration has been reported, including fatal great vessel perforation^17^, ^18^. In addition, the drilling and screwing on the sternal manubrium could increase the risk of mediastinal structures rupturing. For the methods using plates, such as T‐plates and locking compression plates, the plate neither decreases the risk of mediastinal structures rupturing by screwing onto the sternal manubrium nor has any benefit for the movement of the shoulder girdle because the rigid behavior of firm fixation limits the micromotion of sternoclavicular joint^19^, ^20^. Meanwhile, firm fixation of the plate could also increase the risks of internal fixation displacement and breakage, reduction loss, and infection^21^.

In this study, an acromioclavicular joint hook plate was used for the treatment of sternoclavicular joint dislocation. There are some advantages of the treatment using the acromioclavicular hook plate: (i) the hook structure behind the sternal manubrium could reduce the risk of mediastinal structures rupture by screwing; (ii) the acromioclavicular hook plate could offer enough mechanical strength for maintaining the stability of the steroclavicular joint; and (iii) the hook plate could also provide micromotion with a certain range between the hook and sternal manubrium, which could be beneficial for the movement of the shoulder girdle and reduce the risk of displacement and breakage of internal fixation. For the 10 patients in this study, the operations proved safe and effective, with obvious improvement in shoulder girdle movement and no complications.

However, there are still some disadvantages of this treatment: (i) the structure of acromioclavicular joint hook plate is not highly suitable for the anatomical structure of the sternoclavicular joint, and the plate is not in line with the joint; (ii) the hook of the plate is so sharp that there is still some risk of rupture of mediastinal structures; (iii) this hook plate has no special screw hole available for the sternoclavicular joint dislocation with medial clavicle fracture; and (iv) the osteolysis of the sternum caused by the hook plate needs to be observed in further research. Based on these disadvantages, we have designed a new hook plate, for which a Chinese patient has been applied. This new hook plate conforms to the anatomical structure of the sternoclavicular joint, has a blunt hook to avoid rupture risk, and has some screw holes next to the hook for fixing the medial clavicle fracture fragments. In addition, the acromioclavicular joint hook plate has been used for anterior sternoclavicular joint dislocation in this study, but whether it could be used for posterior sternoclavicular joint dislocation should be tested further in the future.

## *Conclusion*

The acromioclavicular joint hook plate demonstrates safety and efficacy for the treatment of anterior sternoclavicular joint dislocation. The hook structure provides micromotion, which is beneficial for the movement of the shoulder girdle, and reduces the risk of mediastinal structure rupture and breakage and displacement of the plate by screwing in the sternal manubrium. However, there are still some disadvantages of this treatment. Further studies are needed to improve the design of the new hook plate in the future.
